# Early Use of Methylene Blue in Vasoplegic Syndrome: A 10-Year Propensity Score-Matched Cohort Study

**DOI:** 10.3390/jcm11041121

**Published:** 2022-02-20

**Authors:** Othmar Kofler, Maximilian Simbeck, Roland Tomasi, Ludwig Christian Hinske, Laura Valentina Klotz, Florian Uhle, Frank Born, Maximilian Pichlmaier, Christian Hagl, Markus Alexander Weigand, Bernhard Zwißler, Vera von Dossow

**Affiliations:** 1 Department of Anesthesiology, Heidelberg University Hospital, Im Neuenheimer Feld 420, 69120 Heidelberg, Germany; florian.uhle@med.uni-heidelberg.de (F.U.); markus.weigand@med.uni-heidelberg.de (M.A.W.); 2 Department of Anesthesiology, Ludwig-Maximilians-University, 80539 Munich, Germany; maximilian.simbeck@hotmail.de (M.S.); roland.tomasi@med.uni-muenchen.de (R.T.); christian.hinske@med.uni-muenchen.de (L.C.H.); bernhard.zwissler@med.uni-muenchen.de (B.Z.); vvondossow@hdz-nrw.de (V.v.D.); 3 Department of Thoracic Surgery, Thoraxklinik Heidelberg, Heidelberg University Hospital, 69120 Heidelberg, Germany; laura.klotz@med.uni-heidelberg.de; 4 Department of Cardiac Surgery, Ludwig-Maximilians-University, 80539 Munich, Germany; frank.born@med.uni-muenchen.de (F.B.); maximilian.pichlmaier@med.uni-muenchen.de (M.P.); christian.hagl@med.uni-muenchen.de (C.H.); 5 Department of Anesthesiology and Pain Therapy, Heart and Diabetes Center Bad Oeynhausen, Ruhr University of Bochum, 44801 Bochum, Germany

**Keywords:** methylene blue, vasoplegic syndrome, vasoplegia, shock, cardiac anesthesia, vasopressin, cardiac surgery, cardiopulmonary bypass

## Abstract

Background: Vasoplegic syndrome is associated with increased morbidity and mortality in patients undergoing cardiac surgery. This retrospective, single-center study aimed to evaluate the effect of early use of methylene blue (MB) on hemodynamics after an intraoperative diagnosis of vasoplegic syndrome (VS). Methods: Over a 10-year period, all patients diagnosed with intraoperative VS (hypotension despite treatment with norepinephrine ≥0.3 μg/kg/min and vasopressin ≥1 IE/h) while undergoing heart surgery and cardiopulmonary bypass were identified, and their data were examined. The intervention group received MB (2 mg/kg intravenous) within 15 min after the diagnosis of vasoplegia, while the control group received standard therapy. The two groups were matched using propensity scores. Results: Of the 1022 patients identified with VS, 221 received MB intraoperatively, and among them, 60 patients received MB within 15 min after the diagnosis of VS. After early MB application, mean arterial pressure was significantly higher, and vasopressor support was significantly lower within the first hour (*p* = 0.015) after the diagnosis of vasoplegia, resulting in a lower cumulative amount of norepinephrine (*p* = 0.018) and vasopressin (*p* = 0.003). The intraoperative need of fresh frozen plasma in the intervention group was lower compared to the control group (*p* = 0.015). Additionally, the intervention group had higher creatinine values in the first three postoperative days (*p* = 0.036) without changes in dialysis incidence. The 90-day survival did not differ significantly (*p* = 0.270). Conclusion: Our results indicate the additive effects of MB use during VS compared to standard vasopressor therapy only. Early MB administration for VS may significantly improve the patients’ hemodynamics with minor side effects.

## 1. Introduction

In cardiac surgery, vasoplegic syndrome (VS) is defined as a vasodilatory shock in the perioperative period and is accompanied by severe hypotension, i.e., therapy-refractory mean arterial pressure (MAP) between 40 and 65 mm Hg and a systemic vascular resistance index (SVRI) between 700 and 1200 dyne × sec × cm^−5^ × m^2^, and normal or elevated cardiac output [1]. The hemodynamics of VS show low wedge and low right atrial pressure [1]. VS was first described by Gomes and colleagues who reported cardioplegia in six cases in Sao Paolo, Brazil, in 1994 [1]. Since then, severe VS has been repeatedly described as a hemodynamic challenge in other diseases, such as septic shock, post-transplantation surgery, burns, anaphylaxis, and trauma [2]. VS occurs as a complication during or after cardiopulmonary bypass (CPB), with an incidence of 5–25%, and causes an increased risk of end organ dysfunction and mortality [3]. Previous studies have reported important risk factors for VS [3,4,5,6], which may result in a systemic inflammatory response syndrome with transient vascular dysfunction refractory to vasopressor therapy [7] and can lead to long-term instability intraoperatively and postoperatively. The pathophysiology of VS is complex and includes a functional dysregulation of smooth vascular muscle cells. In cardiac surgery with CPB, inflammatory mediators lead to adrenoreceptor desensitization and an immediate increase in vasoconstrictive mediators. With the subsequent depletion of the mediators and excess of nitric oxide (NO), dilating mediators predominate and vasoplegic shock persists. NO affects both vasoconstriction and dilation. By activating guanylate cyclase (GC), NO increases cyclic guanosine monophosphate (cGMP) and leads to muscle relaxation. NO also acts through adenosine triphosphate-sensitive potassium channels to inhibit vasoconstriction [8,9]. Therapeutic options in VS include fluid administration and/or vasopressor therapy with catecholamines (first-line therapy with norepinephrine and supplementation with epinephrine) and vasopressin. Modulators of NO and/or inflammation, such as methylene blue (MB), hydroxocobalamin (HY), ascorbic acid, thiamine, and corticosteroids, have been investigated as therapeutic options of VS in several studies [9,10,11,12]. Angiotensin II is the most recently published therapeutic alternative, which was reported to reduce catecholamines for VS [13,14]. The efficacy and efficiency of MB administration for VS during or after CPB has been described by several authors; however, to the best of our knowledge, evidence with larger patient collectives is lacking [15,16,17,18,19]. Previous studies using MB in VS revealed conflicting results, which might have been due to the inclusion of different anesthesiologist-triggered strategies and time-dependent factors. We hypothesized that MB exerts a positive effect in the early stages of severe vasoplegia and can thus prevent secondary complications. Therefore, MB may be useful to treat VS at early stages of the syndrome.

## 2. Materials and Methods

This study was performed in accordance with the Declaration of Helsinki. The study protocol was approved by the Ethics Committee of the Ludwig-Maximilians-University of Munich/Germany (number: 326-16). The need for patient consent was waived because of the retrospective nature of the study.

In this single-center retrospective observational study, data from all patients who developed VS during cardiac surgery with CPB at the LMU Hospital in Munich between 1 April 2006 and 31 March 2016 were reviewed. This period was chosen based on influencing cofactors. Patients who required ≥0.3 µg/kg/min norepinephrine plus vasopressin ≥1 IU/h were considered as having VS. The use of vasopressor agents as a surrogate marker for VS has been described previously [16,20] since invasive hemodynamic values, such as cardiac output and SVRI, were not regularly recorded intraoperatively. Intraoperative continuous esophageal echocardiography ensured the exclusion of cardiogenic shock and confirmed the presence of VS. Patients <18 years old, those undergoing off-pump surgery, those with preoperative venovenous extracorporeal membrane oxygenation or extracorporeal life support system treatment, those with increased preoperative c-reactive protein (CRP), and those without missing data.

The primary outcomes intraoperatively were MAP, fluid administration, and the amount and dose of norepinephrine and vasopressin. Over three days postoperatively, liver function (alanine transaminase) and kidney function (creatinine), as well as CRP and leukocytes, were compared. Mortality was analyzed up to discharge.

Anesthesia was administered according to the Munich cardiac anesthesia standard operating procedure. In brief, patients received oral or intravenous premedication with midazolam (3.75–7.5 mg). Administration of angiotensin converting enzyme-inhibitors and sartane was stopped in elective patients the day before surgery. After the insertion of an arterial line, anesthesia was induced with midazolam, etomidate, or propofol, sufentanil, and rocuronium and maintained with a continuous sufentanil infusion (0.5–1 µg/kg/h) and sevoflurane vaporization (1.5–2.5%). After induction, a central venous catheter and an introducer were inserted to optionally apply a pulmonary artery catheter. The hemodynamic status was monitored intraoperatively by transesophageal echocardiography. For cardioplegia, a crystalloid “Bretschneider” solution (Custodiol^®^, Dr. Köhler Chemie GmbH, Bensheim, Germany) was used. An unfractionated heparin bolus of 400 IU/kg total body weight was injected before CPB initiation followed by additional doses to maintain a target activated clotting time ≥400 s. At the end of the CPB, heparinization was antagonized with a slow protamine infusion. Intraoperative hypotension was treated with the maintenance of isovolemia by fluid boluses and continuous norepinephrine administration. In addition, continuous administration of vasopressin was considered when administering norepinephrine >0.2 µg/kg/min. Additional treatment options were epinephrine to support inotropy and hydrocortisone. MB (2 mg/kg total body weight over an infusion period of 10 min) was considered as a rescue medication in the case of therapy-refractory hypotension, where stable hemodynamics could not be achieved despite continuous norepinephrine administration ≥0.3 µg/kg/min and vasopressin ≥1 IU/h and repetitive norepinephrine boluses by the attending anesthesiologist, independent of the anesthesiologist’s level of training. No repetitive administration of MB was used. After surgery, all patients were sedated, ventilated, transferred to the intensive care unit (ICU), and monitored during the following days. Weaning started after cardiorespiratory stabilization and exclusion of revision triggers.

**Figure 1:**
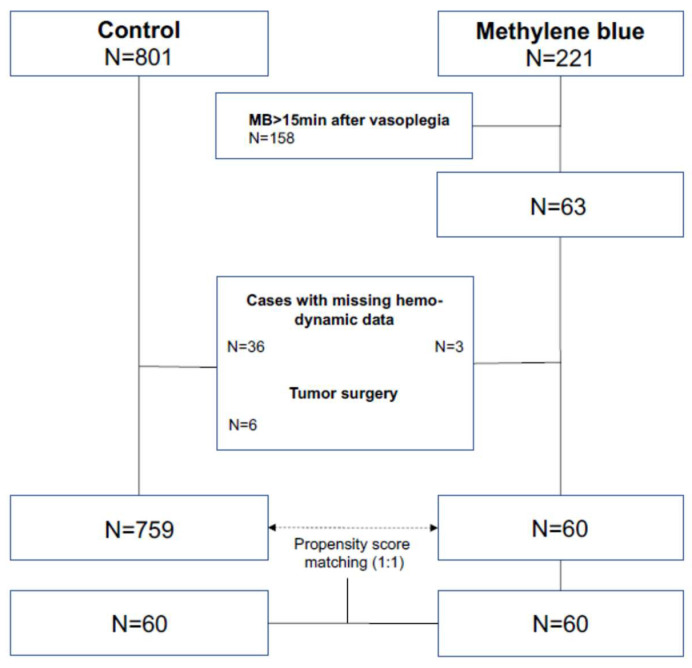
Study population.

After exclusion, patients were divided into three groups based on MB use for hemodynamic rescue from vasoplegia within the first 15 min after the onset of VS (MB group), after 15 min (lMB group), and no MB use (control group, CG). After comparison, the cut off was set to 15 min to evaluate the early effect of MB. Subsequently, the MB group was compared with the CG. Medical records were reviewed to obtain patient demographics and preoperative variables, including sex, age, body mass index (BMI), American Society of Anesthesiologists (ASA) physical classification, surgery type, and emergency status of surgery. For analysis related to the type of surgery, the patients were divided into the following groups: thoracic aortic surgery (aorta), heart valve surgery (valve), isolated coronary artery bypass graft (CABG) surgery (bypass), heart transplantation or ventricular device (artificial heart), combination procedure (e.g., CABG + valve surgery; combination), different types of surgery (e.g., neoplasm; other), revision surgery (revision). To assess the independent effects of early MB on postoperative outcomes, a propensity score-matched analysis was performed. For propensity score matching, the variables age, sex, BMI, and procedure were used. After bivariate analysis (ANOVA) of preoperative factors of all three groups listed in Additional File 1, the propensity for receiving MB variables with a matching tolerance of 0.01 was predicted and included for the procedure. Accordingly, the cases of the MB group were matched 1:1 with corresponding cases of the CG using the propensity score matching function of SPSS^®^ Statistics software (Version 27, IBM Corp., Armonk, NY, USA; Figure 1). This resulted in 60 successfully matched pairs, as evidenced in Table 1.

For intraoperative data collection, the in-house anesthesia recording system NarkoData (IMESO-IT GmbH; Gießen, Germany) was reviewed, and the following variables were analyzed: type of surgery, MAP depending on time since VS (0, +15, +30, +60, +90, +120 min), time-dependent norepinephrine and vasopressin dose and cumulative amount, cumulative fluid administration (crystalloid and colloid) and transfusion needs (erythrocytes, fresh frozen plasma, thrombocytes), duration of surgery, and CPB time. Serum blood samples were routinely taken 24 h preoperatively (not in the case of emergency), on arrival in ICU, and on the first, second, and third postoperative day. Inflammation values (CRP (mg/L), leukocytes (cells/nL)), and values of liver (alanine transaminase (U/L)) and kidney function (creatinine (mg/dL)) were determined for the evaluation of Secondary organ dysfunction. Outcome variables such as ventilation time, in-hospital mortality, length of ICU stay and hospitalization, and postoperative renal replacement therapy were extracted from patient record files.

For continuous variables (e.g., hospitalization), group comparisons were performed using unpaired Student’s *t*-tests. In the case of multiple timepoints, comparisons were individually performed between groups on each timepoint. For categorical variables (e.g., sex), a chi-square test was performed. In the case of two possible conditions, the two-sided Fisher’s exact test *p*-value was reported; for >2 possible conditions, the Pearson’s chi-square *p*-value was reported. Kaplan–Meier analysis was performed for survival time (90 days) with Log-rank group comparison (Mantel Cox). A *p*-value ≤0.05 was considered significant for any comparison.

## 3. Results

During the study period, 1172 out of 9356 patients undergoing cardiac surgery with CPB at this institution were diagnosed with VS, corresponding to an incidence of 12.5%. After the first data validation, 1022 patients were further analyzed. A total of 221 of these patients received MB for hemodynamic rescue from vasoplegia, while 801 patients were not treated with MB and were therefore included in the CG. After excluding patients with missing data and tumor surgery, 759 remained in the CG. The intervention group was then compared with the CG, and the collective was examined for preoperative characteristics. Numerous preoperative and surgical factors were associated with an increased likelihood of receiving MB. The preoperative factors included were older age, higher ASA status, and the type of surgery. Regarding the operative procedure in the non-matched group, patients with thoracic aortic surgery were relatively more likely to receive MB (MB: 26.7 vs. CG: 14.1%), and BMI was significantly correlated with MB treatment (MB: 27.5 vs. CG: 26.2; *p* = 0.024). Emergency surgery status was not correlated with MB treatment (MB: 21.7% vs. CG: 19.9%; *p* = 0.738). To reduce confounding bias, a propensity score-matching analysis was performed, and patients of the MB group were balanced for preoperative covariates. After excluding patients with missing data, 60 patients met the criteria of the MB group. These patients received a bolus of MB within the first 15 min. Demographic and surgery characteristics of the matched cohort are shown in Table 1. Univariate analysis was used to compare the incidence of different intraoperative variables and outcomes in patients who did and did not receive MB (Table 2). The mean surgery duration was >7 h (MB: 421 min ±152 vs. CG: 447 min ±169; *p* = 0.373), and the mean CPB time was approximately 3 h (MB: 183 min ±104 vs. CG: 185 min ±109; *p* = 0.915). We found no significant differences in intraoperative variables.

**Figure 2:**
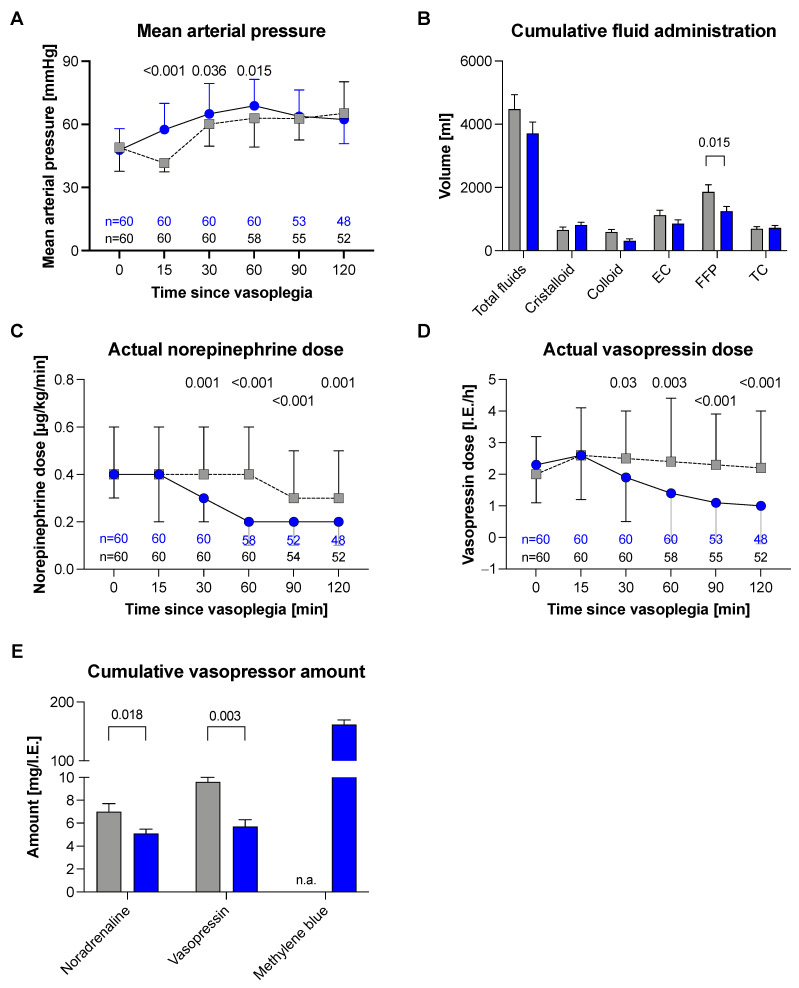
Effects after methylene blue administration in vasoplegic syndrome: The figures show the median (25th–75th percentile) value in the main study group with regard to the use of methylene blue (blue) versus standard therapy (grey). *p*-value indicates standard mean (SD). (**A**) Mean Arterial pressure; (**B**) Cumulative fluid administration; (**C**) Actual norepinephrine dose; (**D**) Actual vasopressin dose; (**E**) Cumulative vasopressoe amount.

MB was administered at a dose of 2 mg/kg total body weight (mean 161.5 mg ± 57.37 mg). The hemodynamic effects compared to the matched pair group are presented in Figure 2. Compared to that in the CG, the MAP in the MB group significantly recovered (Figure 2a) within the first 30 (*p* = 0.036) and 60 min (*p* = 0.015) after diagnosis of VS. Simultaneously, the amount of norepinephrine and vasopressin could be reduced faster in the MB group than in the CG (Figure 2c,d). In addition, the cumulative amount of vasopressors used was lower in the MB group (norepinephrine MB: 7.4 mg ±3.3 vs. CG: 9.7 mg ±6.7; *p* = 0.018 and vasopressin MB: 6.1 IE ±5.1 vs. CG: 11 IE ±13.4, *p* = 0.003; Figure 2e) without the need to substitute more fluids. We only found a difference in the transfusion rates of fresh frozen plasma (MB: 1304 mL ±1200 vs. CG: 2021 mL ±1905, *p* = 0.015; Figure 2c).

**Figure 3:**
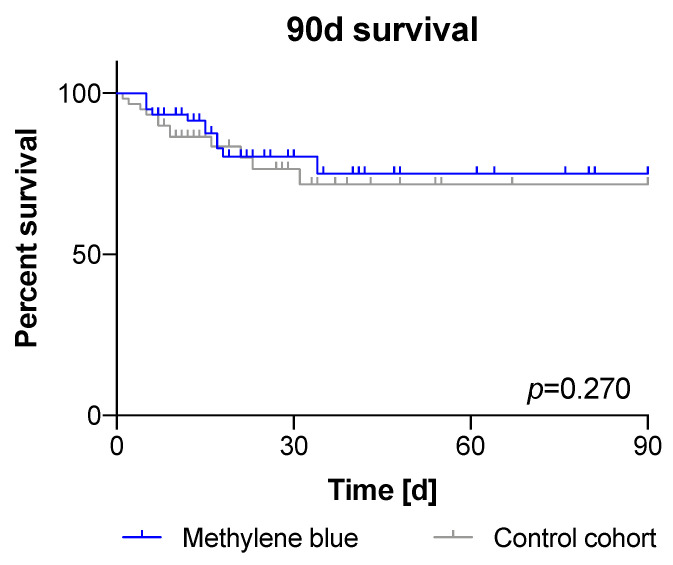
Ninety-day survival after vasoplegic syndrome: The 90-day survival did not differ significantly (MB: 81.7% vs. CG: 80%; *p* = 0.270).

**Figure 4:**
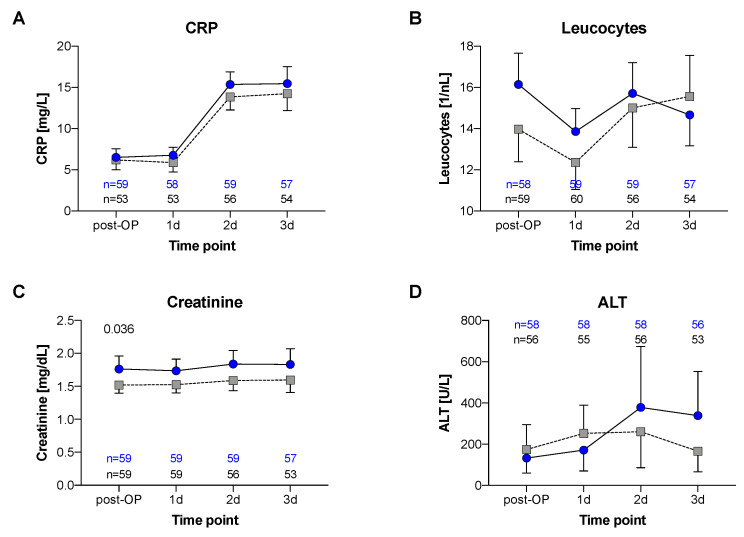
Postoperative variables: Variables are shown with regard to the use of methylene blue versus standard therapy (Matched Control), indicating mean (SD). (**A**) CRP; (**B**) Leucocytes; (**C**) Creatinine; (**D**) ALT.

In addition, the 90-day survival (MB: 81,7% vs. CG: 80%, *p* = 0.270; Figure 3) and other outcome variables did not differ between the groups: mean length of ICU stay (MB: 16 d ±21 vs. CG: 20 d ±37; *p* = 0.466), duration of mechanical ventilation (mean MB: 203 h ±338 vs. CG: 195 h ±275; *p* = 0.918), and length of hospitalization (MB: 30 d ±33 vs. CG: 27 d ±35; *p* = 0.62). In emergency cases, routine blood sampling could not be performed 24 h prior to surgery. Due to this relevant lack of data, the comparison of preoperative values was not meaningful. In the first three postoperative days, CRP and leucocytes did not differ between groups (Figure 4). Regarding comorbidities, we found no higher incidence of liver dysfunction (ALT) in the intervention group, but the MB group was associated with more severe kidney dysfunction (creatinine, *p* = 0.036). Nonetheless, there were no differences in the need of renal replacement therapy (RRT) between groups (27 of 60 patients each group, data not shown).

## 4. Discussion

The current study demonstrated that in our homogenous patient collective, early use of MB after VS diagnosis during cardiac surgery with CPB seems to be associated with beneficial hemodynamic effects compared to the conventional vasopressor support. For MB patients in our series, an improvement in hemodynamic stability within the first hour was associated with a reduction in vasopressor support with norepinephrine and vasopressin. In addition, even if the creatinine values in MB patients were significantly higher in the early postoperative period, the incidence of RRT and postoperative 90-day mortality were not affected.

VS can occur intraoperatively during or after CPB or postoperatively in the ICU [8]. In this study, the overall incidence of VS was 12.5%, which is in accordance with previous reports that show VS occurring among 9–44% of patients undergoing cardiac surgery with CPB [5,21]. VS can last for up to 72 h and is associated with increased mortality of up to 25% [3,21]. Therefore, it is important to recognize VS early and start goal-directed therapy immediately. Fluid administration and vasopressor therapy are considered first-line treatments for VS. Despite the lack of reports showing superiority of one catecholamine over the other, norepinephrine and vasopressin are reported to have positive effects in VS treatment, ensuring adequate perfusion pressure in all organs. Over 20 years ago, Argenziano et al. confirmed MAP increase and catecholamine reduction in VS treatment with vasopressin [22,23]. Therefore, in our institution, vasopressin is used as a second-line option in the case of vasoplegia. Nevertheless, in the case of persistent therapy-refractory VS, further escalation strategies are required.

HY is a potent direct inhibitor of NO and NO synthase and increases the elimination of an endothelial-bound endogenous vasodilator. These mechanisms are probably responsible for HY’ additive effects in VS [10,11,12] and explain why its pharmacological effects differ from those of MB. It is thought that MB inhibits soluble GC by oxidizing the heme domain, thus preventing NO from binding and consequently decreasing the production in cGMP. This mechanism prevents the relaxation of the vascular smooth muscles without directly affecting the different nitric oxide synthase (NOS) isoforms [24,25]. Moreover, MB appears to generate extracellular superoxide anion, which converts NO to nitrate and consequently inhibits vasodilatation [26].

Out of these therapeutic options, different treatment approaches were proposed [9,27,28]. In contrast to previously published treatment regimens [28], Busse et al. recently recommended to start vasopressin administration at lower doses of norepinephrine, followed by MB in cases of therapy-refractory vasoplegia without contraindications.

Our results confirmed the beneficial effects of MB use on hemodynamics without increasing postoperative complications, such as RRT, hepatic injury, and mortality. In contrast, previous studies reported conflicting results regarding the use of MB in VS. While some studies showed decreased cardiac output, reduced renal and hepatic blood flow, higher incidence of arrhythmia, and increased early postoperative mortality after treatment with MB [16,17,18,19], others showed hemodynamic stabilization [18,29,30,31]. VS progresses with an immediate and profound decline in MAP without initial metabolic or organ dysfunction [20]. To prevent organ damage, we consider it crucial to stabilize hemodynamics and reduce the need for catecholamine as soon as possible. In contrast to previous studies, we, therefore, analyzed data of patients with VS who received MB within 15 min after failure of hemodynamic stabilization with data of those who received standard therapy and found that selected patients could benefit from early MB administration. Delayed MB administration after the onset of complications and in combination with NOS and GC capacity exhaustion could be responsible for the higher complication rates in other studies [16]. In addition, other authors emphasized a time-dependent correlation of MB efficacy [19,32,33], wherein MB has the best effect when NOS activity increases and GC is upregulated, that is, within the first eight hours of VS. Therefore, delayed MB administration might have no beneficial effects due to low GC and NOS levels [32,33]. Mehaffey et al. retrospectively compared intraoperative MB treatment for VS after CPB with delayed treatment in the ICU and found that intraoperative administration improved survival and reduced the risk of major adverse events [30]. Again, the results in our high-risk patient collective showed that the vasopressor support was significantly lower with no effect on mortality following the administration of MB within 15 min after the onset of vasoplegia. Therefore, early MB use after VS onset could be a promising therapeutic strategy with low side effects. Prospective analyses are required to confirm these results. The significant difference of fresh frozen plasma substitution between the groups might be caused by the therapeutic attempt of intravascular fluid administration during persistent severe hypotension despite crystalloid infusion and catecholamine support.

Despite MB´s benefits, its contraindications or potential risk factors should always be identified. The use of MB in patients with glucose-6-phosphate dehydrogenase deficiency might cause severe hemolysis [34,35] and existing antidepressant medication could induce serotonin syndrome [36,37]. Additionally, the administration of MB leads to distorted measurements of oxygen saturations during the time of application.

The best dosing regimen for MB is suggested to be a 2 mg/kg total body weight intravenous bolus, followed by a 0.25–2 mg/kg/h continuous infusion, as reported by Evora et al. [19]. At our institution, anesthesiologists administered only an intravenous bolus without continuous infusion, which could be a limitation of this study. Due to the long duration of the study and due to personnel changes in our department during the study period, we think that practitioner effects might be compensated. Nevertheless, this fact has to be addressed in a prospective trial. Another limitation of our study is its single-center and retrospective design. In addition, we did not consider the severity of vasoplegia in our analysis. Intraoperatively, transesophageal echocardiography was used to exclude further impairment of contractility as a cause of hypotension. Within 72 h of arrival at the ICU, there were certain data gaps regarding ICU stay and duration of mechanical ventilation due to the digital documentation. Additionally, no long-term follow-up was performed. The patients included in this investigation are representative of an adult cardiac surgery population admitted at a university hospital. However, we reduced selection bias by utilizing propensity score-matching and analyzing a limited period where MB was administered.

## 5. Conclusions

Early application of MB after the diagnosis of therapy-refractory VS, in our study, was associated with an improvement of hemodynamic stability and reduced vasopressor support within the first hour without increment in fluid administration. In this high-risk patient collective bolus, MB use appears to be safe and seems to have additive effects to standard vasopressor therapy without affecting mortality. Randomized controlled trials are required to confirm our results.

## Figures and Tables

**Table 1:** Baseline demographics of the methylene blue group compared to control group and propensity score-matched control group.

	Methylene Blue N = 60		Control N = 759		*p*-Value	Matched Control N = 60		*p*-Value
Male sex	51	85.0	580	76.4	0.082	44	73.3	0.177
Age (y)	62.3	±12.2	64.0	±13.6	0.351	62.0	±14.2	0.891
BMI (kg/m^2^)	27.5	±4.5	26.2	±4.3	0.024	26.7	±4.8	0.363
ASA class					0.062			0.414
1	0	0	1	0.1		0	0.0	
2	0	0	1	0.1		0	0.0	
3	12	20.0	293	38.6		10	16.7	
4	42	70.0	419	55.2		39	65.0	
5	6	10.0	45	5.9		11	18.3	
Procedure					<0.001			0.031
Aorta	16	26.7	107	14.1		7	11.7	
Valve	21	35.0	302	39.8		20	33.3	
Bypass	8	13.3	225	29.6		18	30.0	
Artificial heart	5	8.3	69	9.1		9	15.0	
Combination	4	6.7	22	2.9		0	0.0	
Other	2	3.3	25	3.3		4	6.7	
Revision	4	6.7	9	1.2		2	3.3	
Emergency	13	21.7	151	19.9	0.738	16	26.7	0.335

Perioperative variables are shown regarding the use of MB versus standard therapy (matched control), indicating mean or percentage, respectively. This table also shows the results compared to the overall collective before matching. *p*-values indicate significance versus “methylene blue” group. BMI: Body mass index; ASA: American Society of Anesthesiologists.

**Table 2:** Perioperative variables of matched participants.

	Methylene Blue N = 60		Matched Control N = 60		*p*-Value
Duration of surgery (min)	421	±152	447	±169	0.373
Bypass duration (min)	183	±104	185	±109	0.915
Duration of mechanical ventilation (h) *	203	±338	195	±275	0.918
Length of hospitalization (d)	30	±33	27	±35	0.620
Length of ICU stay (d) **	16	±21	20	±37	0.466
90-day survival	49	81.7	48	80.0	0.270

Perioperative variables are shown regarding the use of methylene blue versus standard therapy (matched control), indicating mean (SD) or percentage, respectively. *p*-values indicate significance versus “methylene blue” group. ICU: Intensive care unit. *: only data of 25 control cases available, **: only data of 55 control cases available.

## Data Availability

Not applicable.

## Abbreviations

VS—vasoplegic syndrome; MAP—mean arterial pressure; SVRI—systemic vascular resistance index; CPB—cardiopulmonary bypass; NO—nitric oxide; GC—guanylate cyclase; cGMP—cyclic guanosine monophosphate; MB—methylene blue; HY—hydroxocobalamin; BMI—body mass index; ASA—American Society of Anesthesiologists; ICU—intensive care unit; CRP—C-reactive protein; ALT—liver dysfunction; RRT—renal replacement therapy; CG—control group.
